# The Kesty Hyperpigmentation Scale: A Study to Validate a New Tool for Assessing Facial Hyperpigmentation

**DOI:** 10.1111/jocd.70055

**Published:** 2025-04-01

**Authors:** Chelsea E. Kesty, Katarina R. Kesty

**Affiliations:** ^1^ St. Petersburg Skin and Laser St. Petersburg Florida USA; ^2^ Kesty AI St. Petersburg Florida USA

**Keywords:** facial melasma, hyperpigmentation, laser resurfacing, postinflammatory hyperpigmentation, sun damage, sunlight damage

## Abstract

**Background:**

Facial hyperpigmentation due to sun damage, post‐inflammatory hyperpigmentation, and other factors is a common complaint of patients. While lasers and topical treatments are frequently used to manage hyperpigmentation, a standardized way of measuring response to treatment is difficult.

**Aims:**

The Kesty Hyperpigmentation Scale (KHS) is a novel clinical instrument created to provide a consistent approach for evaluating facial hyperpigmentation in both cosmetic dermatology and broader medical settings.

**Methods:**

This study introduces the KHS, describes the process of its creation and validation, and examines its practical uses in clinical settings. Statistical analysis included Gwet's AC2, Kendall's *W*, Spearman's *ρ*/rho, weighted Cohen's kappa, and Bland–Altman analysis.

**Results:**

The findings of the statistical analysis included high ordinal agreement, strong rank concordance, and minimal bias. This supports the conclusion that the novel rating approach is both reliable and valid for assessing skin hyperpigmentation on the given 0–3 scale. The KHS offers an objective framework to measure the severity of hyperpigmentation, helping clinicians track patient progress after cosmetic treatments, and fostering improved communication with patients. Participants in this study found the scale to be user‐friendly, and the majority expressed interest in incorporating it into their practices to document patient conditions.

**Conclusions:**

The KHS is an effective and user‐friendly tool for evaluating facial hyperpigmentation, addressing a significant need within dermatology.

## 1 Introduction

Facial hyperpigmentation is a widespread concern in dermatology, impacting patients with conditions such as melasma, post‐inflammatory hyperpigmentation (PIH), and damage from ultraviolet radiation [1, 2, 3, 4]. In the realm of cosmetic dermatology, hyperpigmentation caused by sun exposure, acne, hormonal imbalances, and other factors is a common complaint during initial patient–doctor consultations [5, 6, 7, 8, 9]. While lasers and topical treatments are frequently used to manage hyperpigmentation, a standardized way of measuring response to treatment is difficult [10, 11, 12, 13, 14, 15]. Existing facial scales often focus on wrinkles, redness, or other cosmetic concerns, making them unsuitable for addressing hyperpigmentation. To address this limitation, the Kesty Hyperpigmentation Scale (KHS) was developed as a five‐point ordinal scale to assess the severity of facial hyperpigmentation (Table 1). This study aimed to validate the KHS through expert review of clinical imagery and assess its utility in practice.

**TABLE 1:** Kesty Hyperpigmentation Scale.

Grade	Description	Examples
0	None: No hyperpigmentation aside from base skin color	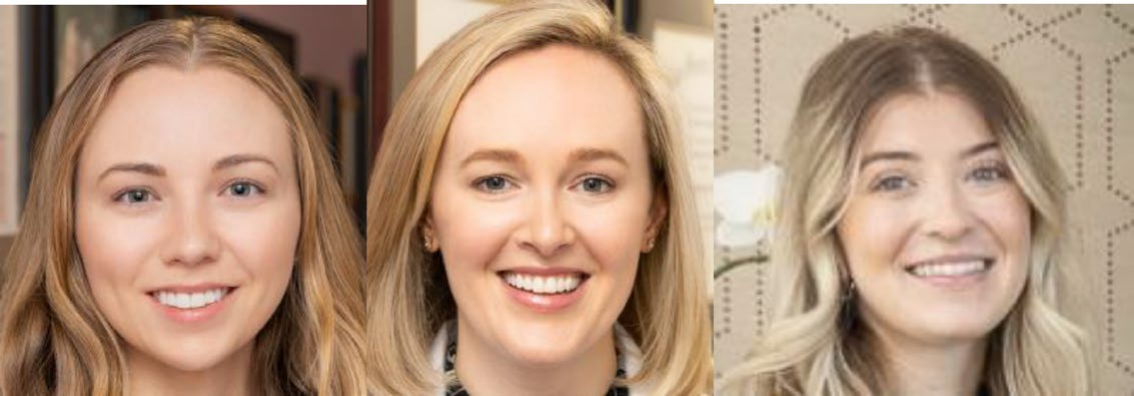
1	Mild: Mildly perceivable brown spots/patch/plaque covering 1%–25% of face	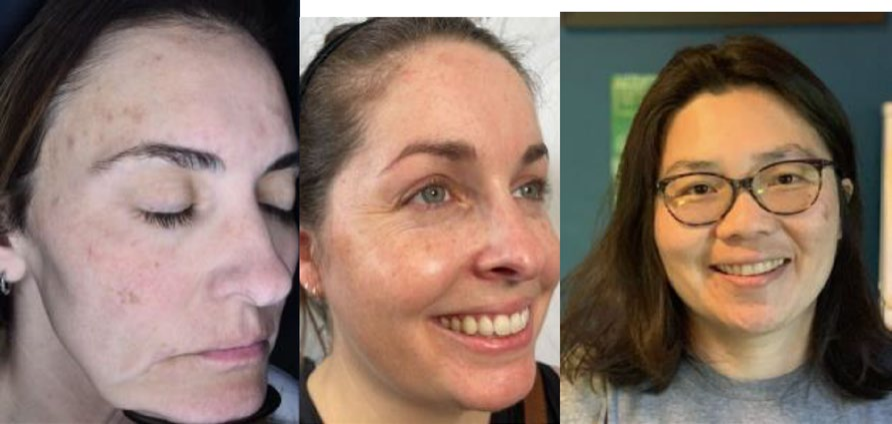
2	Moderate: Moderate brown with 25%–50% face surface area covered with abnormal hyperpigmentation, perceivably uneven skin tone	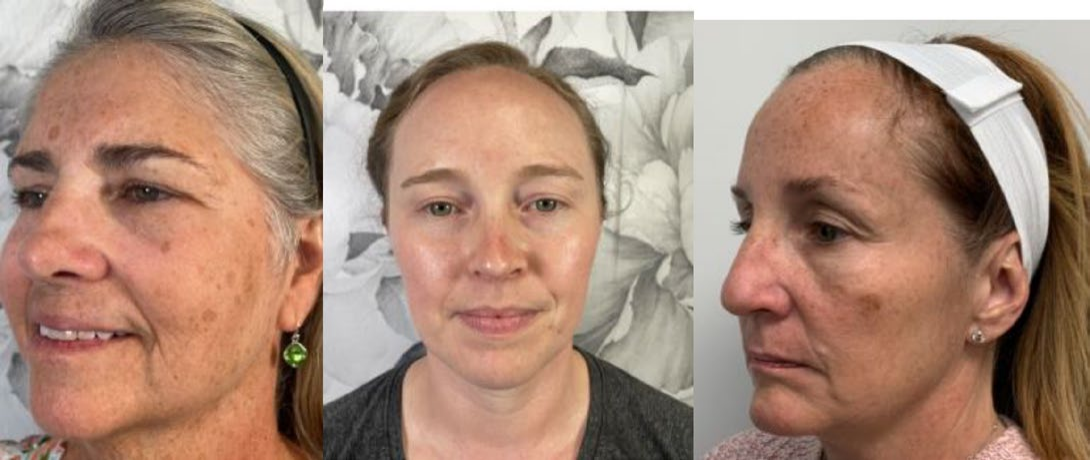
3	Severe: > 50% of face surface area covered with additional pigmentation above base skin color, very easily perceived uneven skin tone	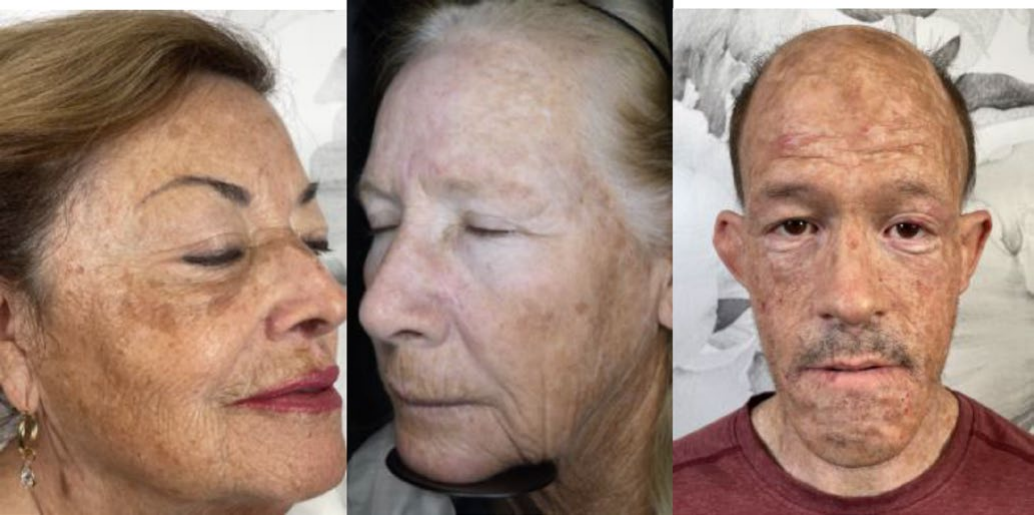

## 2 Methods

A prospective observational study was conducted to evaluate both the inter‐rater reliability and practical application of the KHS. Ten professionals in aesthetic medicine, including board‐certified dermatologists, plastic surgeons, and other aesthetic specialists, served as evaluators. A collection of over 100 facial photographs was assembled from volunteer participants. Each image was taken with the patient either facing forward or slightly turned with both eyes visible. The median age of the photographed subjects was 44 years (range 27–69). The images encompassed varying degrees of hyperpigmentation severity related to aging, photodamage, or dermatological conditions.

A physician‐led study team selected four representative images to correspond with the four severity levels of the KHS. Accompanying written descriptions were also created to define each level (Table 1). The photographs were chosen to demonstrate clear distinctions between the severity levels, with even intervals separating them (e.g., the difference between Level 0 and 1 matched the difference between Levels 1 and 2). This set of reference photographs and their descriptions served as a guide for the evaluators. Alongside these, the evaluators received 20 unlabeled images and were tasked with categorizing them according to the KHS. Additionally, participants responded to two questions: “Is the scale easy to use?” and “Would you utilize this scale in your clinical practice?” (with yes or no as options to respond for each question).

### 2.1 Statistical Methods

In this study, we assessed the reliability and validity of a novel rating method for skin pigmentation (Rater: Kesty) in comparison with multiple expert raters (Raters: B–L). The rating scale was ordinal (0–3). To evaluate both overall and pairwise agreements, we employed a suite of statistical measures tailored to ordinal data. Collectively, the results indicate a strong level of agreement between the novel method and industry professionals.

#### 2.1.1 Overall Measures of Agreement

##### 2.1.1.1 Gwet's AC2

We first estimated Gwet's AC2, a robust, chance‐corrected measure of inter‐rater agreement suitable for ordinal categories. Let the set of categories be $\left\{1,2,\ldots ,R\right\}$ and define quadratic weights as

$$W\left(c_{i},c_{j}\right)=1-{\left(\frac{\left|c_{i}-c_{j}\right|}{R-1}\right)}^{2}$$

The observed agreement $P_{o}$ for N items, each with K raters, is computed by considering category frequencies $f_{c}$ per item and forming pairwise proportions $p_{c_{i},c_{j}}$. Expected agreement $P_{e}$ is derived from the marginal category probabilities $p_{c}$. Gwet's AC2 is given by

$$\mathrm{AC}2=\frac{P_{o}-P_{e}}{1-P_{e}}$$

In our analysis, Gwet's AC2 was approximately 0.9061, indicating excellent agreement.

##### 2.1.1.2 Kendall's *W*

We also computed Kendall's *W*, which measures rank‐based concordance among multiple raters. For *n* items and *m* raters, let $R_{\mathrm{ij}}$ be the rank of the *i*th item by the *j*th rater. Define $R_{i}=\sum_{j=1}^{m}R_{\mathrm{ij}}$ and $\bar{R}=\frac{1}{n}\sum_{i=1}^{n}R_{i}$. Kendall's *W* is given by

$$W=\frac{12\sum_{i=1}^{n}{\left(R_{i}-\bar{R}\right)}^{2}}{m^{2}\left(n^{3}-n\right)}$$

A value of *W* = 0.8551 suggests a high degree of consistency in the ranking of items across raters.

#### 2.1.2 Pairwise Measures of Association and Agreement

##### 2.1.2.1 Spearman's Rank Correlation

To assess the monotonic relationship between the novel method and each expert rater, we employed Spearman's rank correlation (*ρ*/rho). For *n* items, let $d_{i}=R_{i1}-R_{i2}$ be the difference in the ranks assigned by the two raters. Spearman's *ρ*/rho is given by

$$\rho =1-\frac{6\sum_{i=1}^{n}d_{i}^{2}}{n\left(n^{2}-1\right)}$$

Values consistently above 0.90 indicate an extremely strong monotonic relationship (Table 2).

**TABLE 2:** Spearman's rank correlation for the Kesty Hyperpigmentation Scale.

Rater pair	Spearman's *p*
Kesty—B	1.0000
Kesty—C	0.9528
Kesty—D	0.9461
Kesty—E	0.9681
Kesty—F	0.9230
Kesty—G	0.9369
Kesty—H	0.9443
Kesty—I	0.9818
Kesty—J	0.9491
Kesty—K	0.9518
Kesty—L	0.8951

Rater	Bias	Lower limit	Upper limit
B	0.000O	0.0000	0.0000
C	0.2000	−0.6044	1.0044
D	0.0500	−0.7223	0.8223
E	0.1000	−0.5033	0.7033
F	−0.1500	−1.1091	0.8091
G	0.2500	−0.6208	1.1208
H	−0.0500	−0.8223	0.7223
I	0.0500	−0.3883	0.4883
J	0.0500	−0.7223	0.8223
K	−0.0500	−0.8223	0.7223
L	0.0000	−1.1014	1.1014

##### 2.1.2.2 Weighted Cohen's Kappa

As a direct measure of ordinal agreement, we computed the weighted Cohen's kappa. Let $O_{\mathrm{ij}}$ be the observed proportion of assignments where Rater A and another rater choose categories i and j, and let $E_{\mathrm{ij}}$ be the expected proportion under independence. Using the same quadratic weights $w_{\mathrm{ij}}=W\left(c_{i},c_{j}\right)$ defined above, weighted kappa is

$$\kappa_{w}=\frac{\sum_{i,j}w_{\mathrm{ij}}O_{\mathrm{ij}}-\sum_{i,j}w_{\mathrm{ij}}E_{\mathrm{ij}}}{1-\sum_{i,j}w_{\mathrm{ij}}E_{\mathrm{ij}}}$$

Our results showed weighted kappas frequently above 0.90, indicating that the novel method's categorical assignments closely align with the experts (Table 3).

**TABLE 3:** Weighted Cohen's kappa for the hyperpigmentation scale.

Rater pair	Weighted Cohen's kappa
Kesty—B	1.0000
Kesty—C	0.9205
Kesty—D	0.9393
Kesty—E	0.0641
Kesty—F	0.9004
Kesty—G	0.9049
Kesty—H	0.9385
Kesty—I	0.9805
Kesty—J	0.9367
Kesty—K	0.9453
Kesty—L	0.8911

##### 2.1.2.3 Bias and Limits of Agreement

Finally, a Bland–Altman analysis was conducted to explore potential systematic bias. For two raters, define the difference $D_{i}=R_{i1}-R_{i2}$ and the average $A_{i}=\left(R_{i1}+R_{i2}\right)/2$. The mean difference (bias) and the standard deviation (SD) of differences provide limits of agreement (LoA):

$$\text{Bias}=\frac{1}{n}\sum_{i=1}^{n}D_{i},\mathrm{LoA}=\text{Bias}\pm 1.96\cdot \mathrm{SD}$$

Our analysis revealed minimal bias and narrow LoA, suggesting no substantial systematic deviation of the novel method's scores from those of established experts. Although Bland–Altman is more commonly applied to continuous data, it still provides a useful check for consistent over‐ or underestimation, which was not evident here.

## 3 Results

In summary, the combination of Gwet's AC2, Kendall's *W*, Spearman's *ρ*/rho, weighted Cohen's kappa, and Bland–Altman analysis provides a comprehensive view of the novel method's performance. The findings—high ordinal agreement, strong rank concordance, and minimal bias—support the conclusion that the novel rating approach is both reliable and valid for assessing skin hyperpigmentation on the given 0–3 scale. These results position the new method as a credible tool in line with industry standards. 100% of participants responded that this scale was easy to use. All users also stated that they would use this scale as part of their clinical practice.

## 4 Discussion

The demand for cosmetic procedures in the United States has steadily risen, with treatments like lasers for pigmentation correction among the fastest growing segments. Although patient satisfaction is paramount, having an objective tool to measure the outcomes of pigmentation treatments can greatly improve clinician–patient discussions. The KHS was developed to bridge this gap, offering a versatile and standardized approach to evaluating hyperpigmentation.

The KHS has numerous potential applications, including tracking outcomes in treatments such as laser therapy, chemical peels, and prescription topical regimens to treat hyperpigmentation. Clinicians can utilize the scale to document pre‐ and post‐treatment changes, ultimately enhancing patient trust and satisfaction. The scale can also be employed in clinical research as an outcome measure for studies evaluating therapies for melasma, PIH, and photodamage. By simplifying the evaluation process and increasing consistency, the KHS proves effective for both clinical and research settings. Future developments could see the scale incorporated into artificial intelligence platforms, reducing the need for manual assessments.

## 5 Conclusion

The KHS is an effective and user‐friendly tool for evaluating facial hyperpigmentation, addressing a significant need within dermatology. Its validation through expert review and statistical analysis highlights its value for enhancing clinical practice and advancing research. Upcoming investigations could focus on integrating the scale with AI technology to enhance precision and reduce subjectivity in assessments.

## Author Contributions

K.R.K. and C.E.K. conceived the study, wrote and revised the manuscript, and funded the study. All authors have reviewed and approved the article for submission.

## Conflicts of Interest

The authors declare no conflicts of interest.

## Data Availability

The data that support the findings of this study are available on request from the corresponding author. The data are not publicly available due to privacy or ethical restrictions.
